# Prenatal depression exposure and infant developmental outcomes: a retrospective cohort study of reduced fetal growth indicators, elevated neonatal heart rate, and developmental trajectories in China

**DOI:** 10.3389/fpsyt.2026.1827974

**Published:** 2026-06-22

**Authors:** Chen Wang, Shaokai Ning, Xiaoxi Li, Minhui Jiang, Yaling Feng, Qing Xu, Guofu Zhang, Xiaomin Zheng

**Affiliations:** 1 Wuxi Maternity and Child Health Care Hospital (Affiliated Women’s Hospital of Jiangnan University), Wuxi School of Medicine, Jiangnan University, Wuxi, Jiangsu, China; 2 Department of Obstetrics and Gynecology, Women’s Hospital of Nanjing Medical University, Nanjing Women and Children’s Healthcare Hospital, Nanjing, Jiangsu, China; 3 Department of Geriatric Psychiatry, Wuxi Mental Health Center, Wuxi, Jiangsu, China

**Keywords:** infant, neonatal heart rate, prenatal depression, reduced fetal growth indicators, retrospective cohort study, sleep

## Abstract

**Introduction:**

While prenatal depression is recognized as a significant public health issue, its specific impacts on fetal development and subsequent infant growth in the first year of life remain insufficiently characterized in Chinese populations. This study aimed to investigate the effects of prenatal depression exposure on neonatal outcomes and early infancy developmental trajectories.

**Materials and methods:**

This retrospective cohort study utilized data from the Jiangsu Provincial Maternal and Child Health Platform (2006–2023). A total of 146 exposed and 146 control infants were included after 1:1 propensity score matching. After matching and applying exclusion criteria neonatal anthropometric measures (gestational age, birth weight, length, head circumference), pulse rates, and longitudinal growth parameters up to 12 months were analyzed using comparative statistics and regression models. All models were adjusted for key confounders. Missing data were handled by multiple imputation.

**Results:**

Mothers with prenatal depression were younger (mean age 34.01 vs. 36.83 years) and more frequently primiparous. Exposed neonates showed significantly lower but clinically non-pathological anthropometrics: reduced gestational age (38.67 vs. 39.16 weeks, P = 0.001) but still term (≥37 weeks), lower birth weight (3,164.59 vs. 3,356.39 g, P<0.001), shorter body length (49.51 vs. 49.98 cm, P = 0.001), and nominally smaller head circumference (33.61 vs. 33.99 cm, P = 0.028). Substantial missing data limit firm conclusions regarding head circumference. Adverse birth outcomes were more prevalent, with a fourfold higher incidence of low birth weight (12% vs. 3%). Notably, exposed neonates exhibited elevated pulse rates (137.63 vs. 130.6 bpm, P = 0.008), indicative of altered autonomic regulation. Developmental differences persisted into infancy: at six months, exposed infants had lower weight (8.30 vs. 8.48 kg, P = 0.158), shorter length (75.80 vs. 76.13 cm, P = 0.049), and delayed dentition (0.55 vs. 3.86 teeth, P = 0.026), though growth velocity patterns suggested partial catch-up in linear dimensions.

**Conclusions:**

Prenatal depression exposure is associated with lower but clinically non-pathological fetal growth indicators, adverse neonatal outcomes, elevated infant heart rate, and altered early developmental trajectories. Neonatal heart rate may serve as a potential biomarker of prenatal stress. These findings support integrating mental health screening into routine prenatal care and monitoring the early growth and development of exposed infants.

## 1 Introduction

Prenatal depression is a clinically significant psychiatric condition that encompasses depressive episodes occurring before conception or during pregnancy. It is characterized by substantial heterogeneity in etiology, clinical course, and symptom severity (1). Globally, depression during pregnancy affects approximately 10% of pregnant women (2). In China, prenatal depression is clinically diagnosed according to the *Diagnostic and Statistical Manual of Mental Disorders, Fifth Edition* criteria, typically defined as a depressive episode lasting ≥14 days during pregnancy (antenatal depression) (3). Notably, a substantial proportion of postpartum depression cases actually originate in the prenatal period, with longitudinal studies indicating that about 33% emerge during pregnancy and 26% are present before conception (4).

Prenatal depression exposure is associated with a range of adverse pregnancy and infant outcomes, including abnormal fetal heart rate variability (5), impaired infant neurodevelopment (6), preterm birth, low birth weight (7), and later cognitive dysfunction in offspring (8). Proposed mechanisms underlying these associations involve complex biological pathways, such as epigenetic changes, dysregulation of the hypothalamic-pituitary-adrenal axis, and alterations in the gut-brain axis mediated by the gut microbiota.

Despite growing awareness of its public health importance, several critical research gaps remain regarding prenatal depression and its effects on offspring. These include: (1) the need for more precise epidemiological data on depression specifically occurring in the pre-conception and antenatal periods; (2) incomplete understanding of the mechanisms underlying the intergenerational transmission of risks associated with prenatal depression exposure; and (3) the shortage of evidence-based prevention and intervention strategies tailored to mitigate its impact on early child development. To help address these gaps, we established a retrospective cohort study linking maternal demographic data with developmental measures of their offspring during infancy. The primary aim of this study is to systematically examine the impact of prenatal depression exposure on infant growth and developmental outcomes in the first year of life, thereby providing a scientific foundation for developing targeted prevention and management approaches.

## 2 Materials and methods

### 2.1 Study design and participant selection

#### 2.1.1 Study cohort establishment

A population-based retrospective cohort study was conducted using data extracted from the Jiangsu Maternal and Child Health Platform, a comprehensive electronic health record system. The initial study population comprised all mother-infant pairs in the Jiangsu Maternal and Child Health Platform where the infant was born between January 2006 and December 2023. The study protocol was approved by the Ethics Committee of the Affiliated Maternity and Child Health Hospital of Jiangnan University (YLSL2025-068) on September 3rd.

#### 2.1.2 Definition of prenatal depression exposure

Exposure to prenatal depression was defined based on independent clinical diagnostic interviews conducted by psychiatrists or trained obstetricians during routine antenatal care. A positive clinical diagnosis required the presence of DSM-5 Criterion A for a major depressive episode, specifically: (1) persistent depressed mood, and (2) loss of interest or pleasure, lasting for at least two weeks during pregnancy or the pre-conception period.

The Patient Health Questionnaire-9 (PHQ-9) was administered as a quantitative corroborative tool to support clinical assessment, not as the primary definition of exposure. Consistent with routine clinical practice in Chinese perinatal care, a PHQ-9 score of ≥5 was used as the operational threshold to confirm clinical suspicion. This dual-approach definition combines the diagnostic validity of clinical interview with the standardization and reproducibility of a structured questionnaire.

Importantly, exposure classification was driven by the clinical interview findings; the PHQ-9 threshold served only to ensure consistency and quantify symptom burden, not to define caseness.

The PHQ-9 questionnaire was routinely administered to all pregnant women during their first antenatal visit and repeated in each trimester. Exposure timing (first, second, or third trimester) was documented based on the visit when clinical confirmation and PHQ-9 assessment occurred; trimester-specific analyses were not performed due to small subgroup sizes, which is acknowledged as a limitation.

The specific grouping criteria were:

Prenatal Depression-Exposed Group: (1) Gestational age at birth >24 weeks; (2) clinical confirmation of ≥2 weeks of depressed mood and anhedonia based on psychiatric or obstetric interview during any antenatal visit; and (3) a corresponding PHQ-9 score ≥5 at the same visit (or a score ≥2 on item 9 for suicidal ideation, irrespective of total score).

The clinical diagnostic interviews were conducted using a standardized, DSM-5−aligned semi−structured interview protocol for perinatal depression. All assessors (psychiatrists and specialized obstetricians) completed a uniform training program on diagnostic criteria, interview administration, and scoring before study initiation. Inter−rater reliability was formally examined in a pilot sample using Cohen’s kappa coefficient, and excellent inter−rater consistency (κ > 0.85) was achieved, confirming reproducibility of exposure classification.

#### 2.1.3 Control group

Gestational age at birth >24 weeks; no clinical evidence of persistent depressed mood or anhedonia documented during any antenatal visit; and no PHQ-9 score ≥5 recorded at any antenatal visit.

#### 2.1.4 Timing of exposure assessment

Clinical confirmation of prenatal depression and the corresponding PHQ-9 assessment occurred at specific antenatal visits, Exposure was defined as a positive clinical diagnosis at any trimester; trimester-specific analyses were not performed due to insufficient subgroup sample sizes. This limitation is discussed in detail in the Discussion section (see Section 4.5).

#### 2.1.5 Eligibility and exclusion criteria

All mother-infant pairs were required to have the mother aged between 18 and 40 years at delivery and have core infant follow-up data available in the Jiangsu Maternal and Child Health Platform. The following exclusion criteria were applied to both groups: (1) maternal age <18 years or >40 years; (2) pre-conception diagnosis of maternal major depressive disorder or other major psychiatric disorders (e.g., bipolar disorder, schizophrenia); (3) parental history of severe chronic diseases (e.g., cancer, autoimmune diseases) or known congenital genetic disorders diagnosed prior to conception; (4) parental diagnosis of confirmed chromosomal abnormalities or monogenic disorders; (5) infant with major congenital anomalies, chromosomal abnormalities, or severe perinatal complications (e.g., grade III/IV intraventricular hemorrhage, severe hypoxic-ischemic encephalopathy); (6) multiple pregnancies (twins or higher-order multiples); (7) duplicate records for the same infant; and (8) infant deceased before the first postnatal follow-up visit.

**Figure 1:**
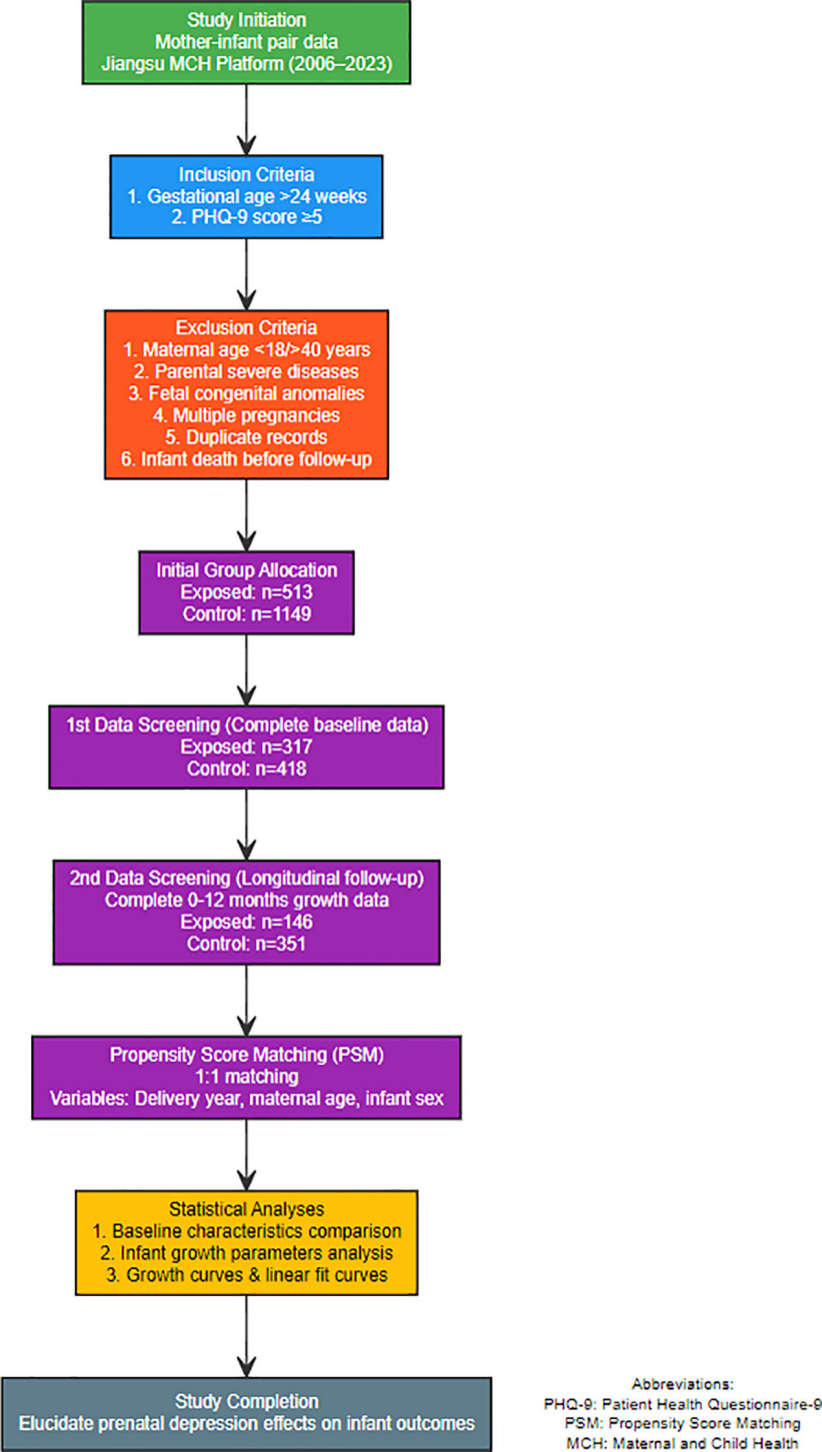
Flowchart of participant selection and cohort derivation for the retrospective cohort study investigating the association between prenatal depression exposure and infant developmental outcomes in a Chinese population.

A total of 1,662 mother-infant pairs were initially identified, and sequential application of inclusion/exclusion criteria and data completeness requirements yielded the final analytic and longitudinal follow-up cohorts (**Figure 1**).

Both full-term (≥37 weeks) and preterm (<37 weeks) infants were included in the study. There was no restriction to very preterm infants (e.g., <32 weeks); the reference to very preterm infants in the literature selection has been corrected to reflect the full range of gestational ages in our cohort (see Section 2.2 for the definition of preterm birth).

### 2.2 Data collection

All data were obtained from structured fields and routine follow-up records within the Jiangsu Maternal and Child Health Platform.

#### 2.2.1 Maternal variables

Data extracted included maternal age at delivery (years), self-reported ethnicity (Han/non-Han), height (cm), pre-pregnancy weight (kg), body mass index (BMI, kg/m²), gravidity, parity, smoking and alcohol use during pregnancy, pregnancy complications, antidepressant use (SSRIs), comorbid anxiety symptoms, breastfeeding status and PHQ-9 screening scores during pregnancy.

#### 2.2.2 Infant variables at birth (0 months)

Sex, gestational age at birth (weeks, based on first-trimester ultrasound), birth weight (grams), birth length (centimeters), and head circumference at birth (centimeters). Neonatal resting heart rate (beats per minute) was recorded as the average of three measurements taken within the first two hours after birth using standardized hospital monitors.

#### 2.2.3 Longitudinal infant follow-up metrics

During scheduled routine health check-ups at 1, 3, 6, 8, and 12 months of age, the following metrics were systematically recorded: anthropometric measurements, including weight (kg), length/height (cm), and head circumference (cm); and developmental and care indicators, where healthcare providers collected parent-reported information on infant daily sleep duration (total hours per 24-hour period) and estimated daily time spent outdoors using structured questionnaires.

Adverse Birth Outcomes Classified as follows:

- Preterm Birth: Delivery <37 completed weeks of gestation (9).
- Small for Gestational Age: Birth weight <10th percentile for gestational age and sex according to INTERGROWTH-21st standards (10).
- Low Birth Weight (LBW): Birth weight <2500 g (11).
- Macrosomia: Birth weight ≥4000 g (11).
- Large for Gestational Age (LGA): Birth weight >90th percentile for gestational age and sex according to INTERGROWTH-21st standards (10).

#### 2.2.4 Justification for adverse outcome classification

While SGA and LGA represent opposite ends of the birth weight spectrum, both are associated with increased perinatal morbidity. SGA infants are at risk for hypoglycemia, hypothermia, and neurodevelopmental delays, while LGA infants face higher risks of birth injury, hypoglycemia, and childhood obesity. Therefore, both extremes were classified as adverse outcomes to capture the full spectrum of growth disturbances associated with prenatal depression.

All mother-infant pairs were extracted from the Jiangsu Provincial Maternal and Child Health (MCH) Platform (2006–2023), with sequential screening based on predefined inclusion/exclusion criteria and data completeness requirements to establish the final analytic cohort and longitudinal follow-up cohort. PHQ-9, Patient Health Questionnaire-9; PSM, Propensity Score Matching.

#### 2.2.5 Data availability for key covariates

The Jiangsu Maternal and Child Health Platform routinely collects data on maternal pre-pregnancy BMI, smoking and alcohol use during pregnancy, and pregnancy complications (e.g., gestational diabetes mellitus, hypertensive disorders of pregnancy). However, preliminary data quality assessment revealed that these variables had substantial missingness (e.g., pre-pregnancy BMI missing in 34% of records; smoking status missing or inconsistently documented in 62%; pregnancy complications fully documented only in a subset of tertiary hospital referrals). Consequently, these variables could not be included as covariates in the primary multivariable regression models without introducing substantial bias or substantially reducing the analytic sample size. The potential impact of this limitation is addressed in the Discussion.

Due to substantial missingness (pre-pregnancy BMI missing in 34% of records; smoking status missing or inconsistently documented in 62%), these variables could not be included as covariates in the primary multivariable regression models. The missing data mechanism and consideration of multiple imputation are discussed in detail in the Limitations section (see Section 4.5).

### 2.3 Statistical analysis

Statistical analyses were performed using R studio (version 4.3.0) and IBM SPSS Statistics (version 25.0). A two-sided p-value <0.05 was considered statistically significant.

#### 2.3.1 Cohort construction and baseline analysis

We performed 1:1 nearest-neighbor propensity score matching without replacement, with a caliper of 0.2 SD of the logit propensity score. The distribution of birth years between the two groups was comparable (P = 0.856), confirming that temporal trends in healthcare practices or socio-environmental factors did not confound the observed associations. After applying all eligibility and exclusion criteria, the final analytic cohort for birth outcome analysis consisted of 317 exposed and 418 non-exposed pairs.

One-to-one nearest-neighbor matching without replacement and with a caliper width of 0.2 standard deviations of the logit of the propensity score was applied. This resulted in a final matched cohort of 146 treated subjects and 146 control subjects. Balance diagnostics showed that the standardized mean difference (SMD) for age improved from 2.701 (indicating severe imbalance) in the unmatched sample to 0.117 in the matched sample (see **Supplementary Figure 1**). Although the post-matching SMD slightly exceeded the conventional threshold of 0.1, it represented a substantial reduction in imbalance. The love plot visualizing this balance improvement is provided in **Supplementary Material 1**. Based on the matched sample, the average treatment effect on the treated (ATT) was 5.01 (95% confidence interval: 2.70 to 7.32), with the associated p-value being less than 0.001.

All analyses were based on this single matched cohort to ensure consistency and reproducibility.

For baseline comparisons, continuous variables were assessed for normality using the Shapiro-Wilk test. Normally distributed data are presented as mean ± standard deviation and compared using independent-sample Student’s t-tests. Non-normally distributed data are presented as median (interquartile range, IQR) and compared using the Mann-Whitney U test. Categorical variables are presented as frequencies (percentages) and compared using Pearson’s chi-square test or Fisher’s exact test, as appropriate.

It should be noted that due to data availability in the primary cohort, the analysis for head circumference at birth included a subset of the final cohort (n=300 exposed and n=79 non-exposed infants). All other analyses were based on the full final cohort unless otherwise specified.

Propensity scores were estimated using logistic regression including delivery year, maternal age, infant sex, ethnicity, gravidity, parity, and gestational age to achieve optimal balance. A 1:1 nearest−neighbor matching without replacement was performed with a caliper of 0.2 SD of the logit propensity score. Standardized mean differences (SMDs) for all matching variables were calculated before and after matching. After matching, all SMDs were <0.10, indicating good balance between groups (see **Supplementary Figure 2**).

#### 2.3.2 Longitudinal data analysis

For the analysis of postnatal growth and developmental trajectories, we included infants who had at least one recorded measurement for anthropometrics, sleep duration, or outdoor time at any of the follow-up time points (1, 3, 6, 8, 12 months). This yielded a longitudinal sub-cohort of 146 exposed and 351 non-exposed infants.

To model the longitudinal trajectories of continuous outcomes (weight, length, head circumference, sleep duration, outdoor time), we employed multilevel regression models (mixed-effects models). These models accounted for the hierarchical structure of the data (repeated measurements nested within infants) and handled unbalanced time points. The models included fixed effects for group (exposed/non-exposed), infant age (modeled as a continuous or categorical variable as appropriate), and their interaction term, along with relevant covariates (e.g. infant sex, gestational age). Random intercepts for each infant were included to account for within-individual correlations. Model assumptions (linearity, homoscedasticity, normality of residuals) were checked graphically.

Missing data were handled using multiple imputation (MI, 5 imputed datasets) under the missing-at-random (MAR) assumption. This approach preserves data variability and reduces bias compared with mean imputation. Complete-case analysis was performed as sensitivity analysis.

#### 2.3.3 Risk factor analysis

To identify independent maternal and neonatal factors associated with prenatal depression exposure, multivariable binary logistic regression was performed. Variables with a univariate association at P<0.10 were considered candidates for the multivariable model, which was built using a backward stepwise selection procedure (likelihood ratio test). Results are reported as adjusted odds ratios (aOR) with 95% confidence intervals.

#### 2.3.4 Multiple comparisons

Benjamini–Hochberg false discovery rate (FDR) correction was applied to control for multiple comparisons across time points and outcome measures. Both unadjusted and adjusted P-values are reported.

## 3 Results

### 3.1 Maternal characteristics and prenatal depression exposure

The final analytic cohort for birth outcomes comprised 317 mother-infant pairs with maternal prenatal depression exposure and 418 non-exposed pairs (**Table 1**).

**Table 1:** Maternal characteristics and demographic profiles.

Variables	Non-depressed	Depressed	*P*-value
Maternal age			
Continuous, mean ± SD (years)	36.83 ± 2.926	34.01 ± 4.662	0.001
Categorical, n (%)			0.001
≤30 years	1	34	
30–40 years	294	91	
≥40 years	56	21	
Parity			
Continuous, mean ± SD	0.48 ± 0.609	0.37 ± 0.555	0.001
Categorical, n (%)			0.481
0	201	90	
1	128	43	
2	18	2	
>2	1	1	
Maternal ethnicity, n (%)			0.657
Han nationality	347	142	
Ethnic minorities	4	1	
History of previous childbirth, n (%)			0.089
Yes	201	90	
No	147	46	
Breastfeeding status, n (%)			0.001
Yes	266	93	
No	85	53	
Continuous indicators, mean ± SD			
Maternal height (cm)	162.3 ± 5.2	161.8 ± 5.6	0.215
Pre-pregnancy weight (kg)	58.4 ± 8.7	57.9 ± 9.1	0.478
Pre-pregnancy BMI (kg/m²)	22.1 ± 3.2	22.0 ± 3.4	0.702

Continuous variables are presented as mean ± standard deviation and were compared using the Student’s *t*-test. Categorical variables are presented as number (percentage).

Mothers with prenatal depression were significantly younger (mean age: 34.01 ± 4.66 vs. 36.83 ± 2.93 years, p<0.001) and had lower parity (0.37 ± 0.56 vs. 0.48 ± 0.61, p=0.001) compared to non-depressed mothers. No significant difference was observed in maternal ethnicity between the two groups (p=0.657). Breastfeeding initiation rate was lower in the prenatal depression group (63.7% vs. 75.8%, p<0.001).

### 3.2 Impact of prenatal depression exposure on neonatal birth outcomes

To delineate the specific effects of prenatal depression on neonatal health, we analyzed key birth parameters from our retrospective cohort.

#### 3.2.1 Reduced gestational age and neonatal anthropometric differences

Infants exposed to maternal prenatal depression exhibited significant reductions in key growth indicators at birth (**Table 2**). Compared to the non-exposed group, the exposed group had a shorter mean gestational age (38.67 ± 1.60 vs. 39.16 ± 1.28 weeks, P = 0.001). Both groups delivered at term (≥37 weeks), and this difference does not indicate preterm birth.

**Table 2:** Newborn characteristics and adverse pregnancy outcomes.

Variables	Non-depressed	Depressed	*P*-value
Gestational age, mean ± SD (days)	39.16 ± 1.275	38.67 ± 1.601	0.001
Newborn gender, n (%)			0.103
Male	194	69	
Female	157	77	
Birth length, mean ± SD (cm)	49.98	49.51	0.001
Birth weight, mean ± SD (g)	3356.39	3164.59	0.001
Birth head circumference, mean ± SD (cm)	33.99	33.61	0.028
Newborn heart rate, mean ± SD (beats/min)			
Male	131.45	138.91	0.011
Female	129.75	136.34	0.005

Continuous variables are presented as mean ± standard deviation and were compared using the Student’s t-test. Categorical variables are presented as number (percentage). NS = Not significant. Head circumference analysis included a subset of infants (n=300 exposed, n=79 non-exposed) due to extensive missing data; results should be interpreted with caution.

Exposed neonates showed significantly lower birth weight (3164.59 ± 466.12 vs. 3356.39 ± 411.33 g, P < 0.001), shorter birth length (49.51 ± 2.01 vs. 49.98 ± 1.85 cm, P < 0.001), and nominally smaller head circumference (33.61 ± 1.40 vs. 33.99 ± 1.25 cm, P = 0.028). All mean values remained within normal clinical ranges, and these anthropometric differences do not meet diagnostic criteria for fetal growth restriction (FGR) or small for gestational age (SGA). Head circumference was analyzed in a limited subset (n=300 exposed, n=79 non−exposed) due to extensive missing data, which precludes firm conclusions on this measure (12).

The exposed group had a fourfold higher incidence of low birth weight (<2500 g) compared with controls (12% vs. 3%), representing a clinically relevant adverse outcome. In contrast, prenatal depression exposure showed no significant effect on infant sex distribution (P = 0.103).

#### 3.2.2 Elevated neonatal heart rate

Notably, exposed neonates exhibited exploratory evidence of elevated pulse rates (137.63 vs 130.6 bpm, P = 0.008). This elevation was consistent in both sexes (males: 138.91 vs. 131.45 bpm, P = 0.011; females: 136.34 vs. 129.75 bpm, P = 0.005). Sleep duration was significantly longer in the exposed group at 8 and 12 months (both P<0.05). These findings should be considered exploratory given the single-time-point measurement and lack of adjustment for delivery mode and acute peripartum stress.

#### 3.2.3 Increased incidence of low birth weight and no effect on sex distribution

The clinical impact of these growth differences was reflected in a fourfold higher incidence of low birth weight (<2500 g) in the exposed group (12% vs. 3%). In contrast, prenatal depression exposure showed no significant effect on infant sex distribution (P = 0.103).

### 3.3 Longitudinal trajectories of infant growth and sleep patterns up to 12 months

Longitudinal data on weight, length, head circumference, and sleep duration were collected from 146 infants exposed to prenatal depression and 351 non-exposed control infants at 1, 3, 6, 8, and 12 months of age. Group differences were analyzed at each time point.

#### 3.3.1 Postnatal growth trajectories and evidence of catch-up growth

Infants exposed to prenatal depression exhibited distinct postnatal growth patterns, with initial deficits showing partial attenuation over time (**Table 3**). Although exposed infants had significantly lower birth weight and sustained this deficit at 1 month (4.45 vs. 4.79 kg, P<0.001), longitudinal mixed-effects models indicated with weight differences becoming statistically non-significant from 3 months onward (P>0.05).

**Table 3:** Sleep duration, body weight and height in infants aged 1–12 months: a comparative analysis.

Time		Non depressed	Depressed	P-value
1 month	Sleep Time	15.81	15.70	0.915
	Weight	4.79	4.45	0.000
	Height	55.26	54.22	0.000
	head circumference	37.46	36.87	0.000
3 months	Sleep Time	13.53	15.15	0.097
	Weight	6.84	6.75	0.277
	Height	62.37	61.83	0.020
	head circumference	40.48	40.26	0.096
6 months	Sleep Time	13.18	14.15	0.177
	Weight	8.48	8.30	0.158
	Height	68.39	67.91	0.040
	head circumference	43.19	43.01	0.170
8 months	Sleep Time	12.00	13.55	0.042
	Weight	9.13	8.96	0.112
	Height	71.55	70.87	0.004
	head circumference	44.41	44.26	0.254
12 months	Sleep Time	11.35	13.02	0.016
	Weight	10.76	10.01	0.114
	Height	76.13	75.80	0.499
	head circumference	45.94	45.72	0.370

Continuous variables are presented as mean ± standard deviation and were compared using the Student’s *t*-test. Categorical variables are presented as number (percentage). NS = Not significant. Sleep duration is measured in hours(h); Body weight is measured in kilograms(Kg); Body height is measured in centimeters(cm) head circumference is measured in centimeters(cm). Some data may be missing, and the missing data will not be included in the statistics.

A similar pattern was observed for linear growth. Exposed infants had significantly shorter length at 1, 3, 6, and 8 months (P<0.05). However, by 12 months, this difference was no longer significant (75.80 vs. 76.13 cm, P = 0.499), indicating a catch-up growth pattern in body length during the latter half of the first year.

Head circumference also showed early deficits in the exposed group at 1 month (36.87 vs. 37.46 cm, P = 0.001). While mean values remained slightly smaller throughout the observation period, statistical significance diminished after the neonatal phase (Δ=0.39-0.61cm, P>0.05), paralleling the trajectory observed for weight.

#### 3.3.2 Prolonged sleep duration in exposed infants

Sleep patterns differed significantly between groups during later infancy. Although no significant difference in sleep duration was observed at birth (15.81 vs. 15.70 hours, P = 0.915), exposed infants consistently showed longer sleep durations as they aged. A statistically significant difference emerged at 8 months (13.55 vs. 12.00 hours, P = 0.042) and persisted at 12 months (13.02 vs. 11.35 hours, P = 0.016).

### 3.4 Growth velocity patterns and maternal risk factors

#### 3.4.1 Differential growth velocities following prenatal depression exposure

**Figure 2:**
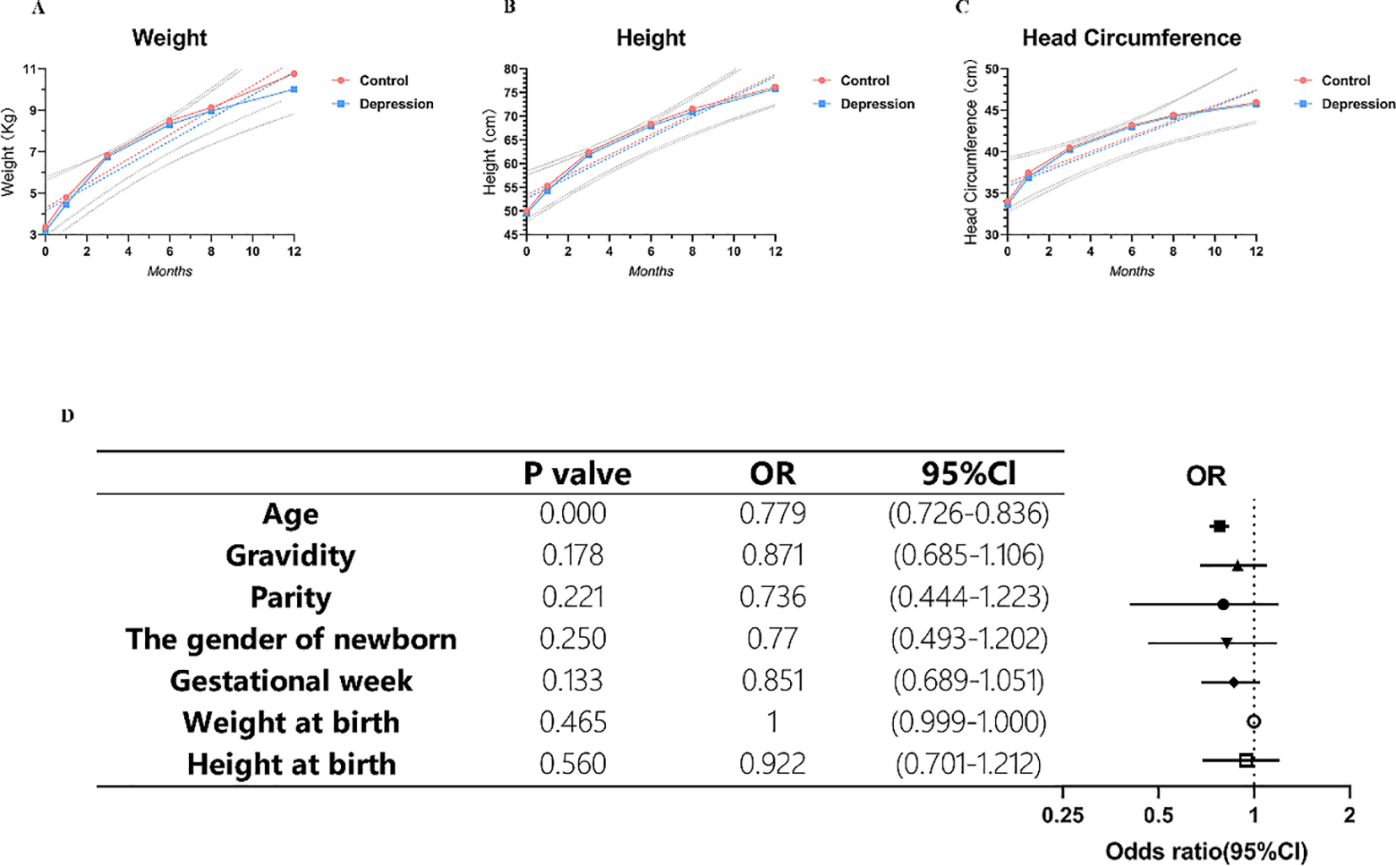
Linear regression curves and multivariate analysis of infant growth parameters associated with prenatal depression exposure. **(A)** Linear regression curve for weight from 1 to 12 months of age. **(B)** Linear regression curve for length from 1 to 12 months of age. **(C)** Linear regression curve for head circumference from 1 to 12 months of age. **(D)** Forest plot of multivariable logistic regression analysis for independent risk factors associated with prenatal depression exposure (χ² = 56.253, P < 0.001).

Analysis of growth velocity patterns revealed distinct postnatal trajectories (**Figures 2A–C**). Infants exposed to prenatal depression demonstrated significantly faster growth velocities in body length and head circumference compared to non-exposed infants, indicating a clear pattern of postnatal catch-up growth following. lower prenatal growth indicators In contrast, weight gain velocity in the exposed group remained persistently slower throughout the first year of life. These differential velocity patterns across anthropometric parameters suggest that the effects of prenatal depression exposure on postnatal growth dynamics are domain-specific.

#### 3.4.2 Maternal age as a risk factor for prenatal depression

Multivariable logistic regression analysis was performed to identify factors associated with prenatal depression exposure, adjusting for maternal age, gravidity, parity, infant sex, gestational age, birth weight, and birth length (**Figure 2D**). Younger maternal age was significantly associated with increased odds of prenatal depression exposure (adjusted odds ratio per year increase = 0.779, 95% CI: 0.726–0.836, P < 0.001). No other variables in the model showed statistically significant independent associations.

It should be noted that several important potential confounders—including maternal pre-pregnancy BMI, smoking and alcohol use during pregnancy, and pregnancy complications—could not be adjusted for due to high rates of missing data in the electronic health records (see Section 2.2). The potential impact of this limitation on the observed association between maternal age and depression is examined in the Discussion.

## 4 Discussion

Accumulating evidence has confirmed that adverse intrauterine psychological exposure profoundly programs offspring postnatal growth and developmental phenotypes (13). The biopsychosocial framework underscores the integral role of prenatal mental health in maternal-infant physiology and developmental outcomes (14). Prenatal depression not only impairs maternal well-being but also poses significant risks to fetal and infant development (15). While data on prenatal depression in China remain fragmented (16), large-scale studies investigating its specific intergenerational effects are limited. Our retrospective cohort study addresses this gap by examining the associations between prenatal depression exposure and a spectrum of infant outcomes, providing evidence to inform targeted prevention and clinical monitoring strategies.

### 4.1 Maternal characteristics, breastfeeding initiation, and early care

Our findings indicate that prenatal depression is associated with distinct maternal characteristics and early caregiving practices. Mothers with prenatal depression in our cohort were significantly younger and had lower parity, aligning with global evidence identifying younger age as a key demographic risk factor for antenatal mood disorders (17). Furthermore, we observed a substantially lower rate of breastfeeding initiation among mothers with prenatal depression (63.7% vs. 75.8% in controls). This suggests that depressive symptoms, such as anhedonia or challenges in maternal role adaptation, may adversely affect early feeding motivation and mother-infant interaction (18). These findings highlight a critical postnatal window for providing targeted support to facilitate bonding and promote optimal nutritional practices for vulnerable mother-infant dyads.

### 4.2 Neonatal outcomes: lower anthropometrics and altered physiological state

This study provides clear evidence linking maternal prenatal depression to moderately lower but clinically non-pathological neonatal anthropometrics rather than overt fetal growth restriction. Infants exposed to prenatal depression exhibited significant reductions in birth weight, length, and head circumference without meeting clinical definitions of FGR, SGA, or microcephaly. The fourfold higher incidence of low birth weight (<2500 g) in the exposed group (12% vs. 3%) underscores a tangible clinical risk, consistent with meta-analytic evidence associating maternal depression with adverse birth weight outcomes.

Head circumference results should be interpreted with extreme caution due to large amounts of missing data in one group, which limits the reliability and generalizability of this comparison.

A notable physiological finding was the significantly elevated resting heart rate observed in exposed neonates, evident in both sexes. This suggests a potential dysregulation of the autonomic nervous system, possibly originating from maternal stress-induced activation of the hypothalamic-pituitary-adrenal axis and consequent fetal exposure to elevated glucocorticoids (19). Although elevated neonatal heart rate has been linked to uteroplacental insufficiency and chronic prenatal stress (20), the single measurement taken shortly after birth cannot fully exclude the influence of peripartum events, such as mode of delivery (e.g., cesarean section vs. vaginal delivery), neonatal pain response, or low Apgar scores, which may independently elevate neonatal heart rate. Given the single postnatal measurement, lack of adjustment for delivery−related factors, and potential influence of acute neonatal stress, elevated heart rate should be regarded as an exploratory indicator of prenatal stress exposure rather than a validated biomarker.

### 4.3 Infant growth trajectories and sleep patterns in the first year

Longitudinal assessment revealed distinct developmental trajectories during infancy. While infants exposed to prenatal depression showed initial deficits in weight, length, and head circumference, growth velocity analysis indicated a pattern of partial catch-up, particularly in linear growth (body length). By 12 months of age, the significant deficit in length had resolved, a phenomenon observed in other contexts of early growth compromise (21). In contrast, weight gain velocity remained persistently slower in the exposed group throughout the first year, suggesting that the effects of prenatal depression on postnatal growth may involve domain-specific metabolic or nutritional pathways.

Additionally, we identified significantly prolonged sleep duration in exposed infants from 8 months of age onward. Altered sleep architecture in infancy has been linked to subsequent neurodevelopmental profiles (22). This finding may reflect either a compensatory need for neural rest or differences in circadian regulation associated with prenatal depression exposure, meriting further exploration as a potential early-life indicator of altered developmental trajectories.

### 4.4 Younger maternal age as a key modifiable risk factor

Our multivariable analysis identified younger maternal age as the sole independent factor significantly associated with prenatal depression exposure (adjusted OR per year increase = 0.779, 95% CI: 0.726–0.836), corroborating international research on age-related vulnerability (23). This translates to a 22% reduction in the odds of depression exposure with each increasing year of maternal age. This finding carries direct clinical relevance: maternal age can function as a simple, readily available factor for initial risk stratification in prenatal care settings. Implementing routine, validated mental health screening (e.g., Patient Health Questionnaire-9) during antenatal visits, with particular attention to younger expectant mothers, represents a feasible and proactive strategy for early identification and intervention (24).

### 4.5 Limitations

Several limitations of this study should be considered when interpreting the findings.

Limitations regarding unmeasured confounding: Our analyses could not adjust for several well-established confounders of the association between prenatal depression and infant outcomes, including maternal pre-pregnancy BMI, smoking and alcohol use during pregnancy, and pregnancy complications (e.g., GDM, preeclampsia). These variables were either missing at high rates or inconsistently documented in the Jiangsu Maternal and Child Health Platform, a limitation inherent to retrospective analyses of routinely collected electronic health data. This limitation is non-trivial: maternal obesity (high BMI) is independently associated with both depression and increased birth weight; smoking is associated with both depression and reduced birth weight; and pregnancy complications such as GDM and preeclampsia are risk factors for both maternal mood disorders and adverse neonatal outcomes. The absence of adjustment for these factors may introduce residual confounding, potentially biasing our effect estimates either toward or away from the null. For example, if smoking prevalence were higher in the depression-exposed group (as suggested by literature), the observed associations between depression and reduced birth weight could be partially or fully confounded by smoking. Conversely, if obesity were more common in the exposed group, it might attenuate observed differences in birth weight. Readers should therefore interpret our findings as associations rather than causal effects, and the results should be considered hypothesis-generating rather than confirmatory. Future prospective studies with systematic collection of these covariates are needed to validate and extend our findings.

Missing data mechanism and handling: Missing covariates followed a missing at random (MAR) mechanism related to documentation practices. Although multiple imputation was evaluated, the high proportion of missing data limited its utility. Residual confounding therefore remains an important limitation, and findings should be interpreted as associational rather than causal.

Limitation regarding the timing of prenatal depression exposure: Due to limited sample size, we were unable to perform trimester-stratified analyses to examine whether the association between prenatal depression and infant outcomes varies by the gestational timing of exposure. This is a non-trivial limitation because fetal neurological and physical development shows differential sensitivity to maternal stress depending on gestational age. For instance, depression in the first trimester may primarily affect embryogenesis and organ formation, whereas depression in the third trimester may more directly influence autonomic nervous system regulation (e.g., neonatal heart rate variability). By aggregating all exposure periods into a single “any trimester” category, our analysis may obscure these trimester-specific mechanisms and potentially misestimate the true effects of prenatal depression. Future prospective studies with larger sample sizes are needed to disentangle the distinct developmental consequences of depression occurring in each trimester.

Limitation regarding sleep measurement: Infant sleep duration was assessed using parent-reported 24-hour recall without objective validation (e.g., actigraphy or sleep diaries), which may introduce recall bias and social desirability bias. Additionally, sleep duration was reported only as total hours per 24-hour period, with no distinction between daytime sleep (naps) and nighttime sleep. This aggregation prevented analysis of important dimensions of sleep architecture, such as sleep fragmentation, frequency of nocturnal awakenings, and the distribution of sleep across day and night—all of which may be differentially affected by prenatal depression exposure and carry distinct developmental implications. Future studies employing objective sleep measurement tools (e.g., actigraphy) and more detailed sleep diaries are needed to validate and extend our findings.

Additionally, maternal antidepressant use (e.g., SSRIs) during pregnancy, which may independently influence neonatal head circumference and sleep patterns, was not adjusted for due to low documented prevalence (<5% in the exposed group) and insufficient sample size for meaningful analysis. Future studies with larger samples should account for medication effects.

## 5 Conclusion

Prenatal depression exposure is associated with a measurable cascade of effects, spanning from reduced fetal growth indicators and altered neonatal physiology to distinct patterns of postnatal growth and sleep in early infancy. Younger maternal age represents a significant and independent risk factor. These findings underscore the necessity of integrating routine mental health screening into standard perinatal care in China. Systematic monitoring of key infant parameters—such as heart rate at birth and longitudinal growth and sleep patterns—may aid in the early identification of offspring at heightened developmental risk. Future prospective studies should combine detailed maternal phenotyping with longer-term developmental assessments in children to further elucidate underlying mechanisms and optimize targeted early interventions.

## Data Availability

The original contributions presented in the study are included in the article/**Supplementary Material**. Further inquiries can be directed to the corresponding authors.

## References

[1] Stewart AL Payne JL . Perinatal depression: a review and an update. Psychiatr Clinics North America. (2023) 46:447–61. doi: 10.1016/j.psc.2023.04.003 37500243

[2] Liu Q He H Yang J Feng X Zhao F Lyu J . Changes in the global burden of depression from 1990 to 2017: findings from the Global Burden of Disease study. J Psychiatr Res. (2020) 126:134–40. doi: 10.1016/j.jpsychires.2019.08.002 31439359

[3] Feng Y Xiao L Wang WW Ungvari GS Ng CH Wang G . Guidelines for the diagnosis and treatment of depressive disorders in China: the second edition. J Affect Disord. (2019) 253:352–6. doi: 10.1016/j.jad.2019.04.104 31078835

[4] Wisner KL Sit DK McShea MC Rizzo DM Zoretich RA Hughes CL . Onset timing, thoughts of self-harm, and diagnoses in postpartum women with screen-positive depression findings. JAMA Psychiatry. (2013) 70:490–8. doi: 10.1001/jamapsychiatry.2013.87 23487258 PMC4440326

[5] Pinto TM Nogueira-Silva C Figueiredo B . Fetal heart rate variability and infant self-regulation: the impact of mother's prenatal depressive symptoms. J Reprod Infant Psychol. (2023) 43:1–14. doi: 10.1080/02646838.2023.2257730 37726914

[6] Fernandes M Srinivasan K Menezes G Ramchandani PG . Prenatal depression, fetal neurobehavior, and infant temperament: novel insights on early neurodevelopment from a socioeconomically disadvantaged Indian cohort. Dev Psychopathol. (2018) 30:725–42. doi: 10.1017/s0954579418000615 30068420

[7] Loret de Mola C de França GV Quevedo LA Horta BL . Low birth weight, preterm birth and small for gestational age association with adult depression: systematic review and meta-analysis. Br J Psychiatry: J Ment Sci. (2014) 205:340–7. doi: 10.1192/bjp.bp.113.139014 25368358

[8] Price RB Duman R . Neuroplasticity in cognitive and psychological mechanisms of depression: an integrative model. Mol Psychiatry. (2020) 25:530–43. doi: 10.1038/s41380-019-0615-x 31801966 PMC7047599

[9] Mikhova M Ivanov S . Clinical and paraclinical markers in early diagnosis of premature birth. Akusherstvo i Ginekologiia. (2005) 44:29–32. 16032910

[10] Villar J Cheikh Ismail L Victora CG Ohuma EO Bertino E Altman DG . International standards for newborn weight, length, and head circumference by gestational age and sex: the Newborn Cross-Sectional Study of the INTERGROWTH-21st Project. Lancet. (2014) 384:857–70. doi: 10.1016/s0140-6736(14)60932-6 25209487

[11] Slancheva B Mumdzhiev H . Small for gestational age newborns--definition, etiology and neonatal treatment. Akusherstvo i Ginekologiia. (2013) 52:25–32. 23807977

[12] Moore ER Bergman N Anderson GC Medley N . Early skin-to-skin contact for mothers and their healthy newborn infants. Cochrane Database Systematic Rev. (2016) 11:Cd003519. doi: 10.1002/14651858.cd003519.pub2 27885658 PMC6464366

[13] Zou R Tiemeier H van der Ende J Verhulst FC Muetzel RL White T . Exposure to maternal depressive symptoms in fetal life or childhood and offspring brain development: a population-based imaging study. Am J Psychiatry. (2019) 176:702–10. doi: 10.1176/appi.ajp.2019.18080970 31055967

[14] Christian P . Micronutrients, birth weight, and survival. Annu Rev Nutr. (2010) 30:83–104. doi: 10.1146/annurev.nutr.012809.104813 20415580

[15] Molani-Gol R Alizadeh M Kheirouri S Hamedi-Kalajahi F . The early life growth of head circumference, weight, and height in infants with autism spectrum disorders: a systematic review. BMC Pediatr. (2023) 23:619. doi: 10.1186/s12887-023-04445-9 38066466 PMC10704616

[16] Fleming S Thompson M Stevens R Heneghan C Plüddemann A Maconochie I . Normal ranges of heart rate and respiratory rate in children from birth to 18 years of age: a systematic review of observational studies. Lancet (London England). (2011) 377:1011–8. doi: 10.1016/s0140-6736(10)62226-x 21411136 PMC3789232

[17] Iseger TA Vollebregt MA Krepel N Arns M . Heart rate variability related to season of birth: a replication study. Psychophysiology. (2019) 56:e13419. doi: 10.1111/psyp.13419 31206750 PMC6852341

[18] Ingemarsson I Herbst A Thorngren-Jerneck K . Long term outcome after umbilical artery acidaemia at term birth: influence of gender and duration of fetal heart rate abnormalities. Br J Obstetrics Gynaecology. (1997) 104:1123–7. doi: 10.1111/j.1471-0528.1997.tb10934.x 9332988

[19] Galland BC Taylor BJ Elder DE Herbison P . Normal sleep patterns in infants and children: a systematic review of observational studies. Sleep Med Rev. (2012) 16:213–22. doi: 10.1016/j.smrv.2011.06.001 21784676

[20] Engel GL . The need for a new medical model: a challenge for biomedicine. Sci (New York NY). (1977) 196:129–36. doi: 10.4135/9781446221129.n3 847460

[21] Rollè L Giordano M Santoniccolo F Trombetta T . Prenatal attachment and perinatal depression: a systematic review. Int J Environ Res Public Health. (2020) 17:2644. doi: 10.3390/ijerph17082644 32290590 PMC7216181

[22] Hagatulah N Bränn E Oberg AS Valdimarsdóttir UA Shen Q Lu D . Perinatal depression and risk of mortality: nationwide, register based study in Sweden. BMJ (Clinical Res Ed). (2024) 384:e075462. doi: 10.1097/01.ogx.0001027444.47162.09 38199643 PMC10777893

[23] Nisar A Yin J Waqas A Bai X Wang D Rahman A . Prevalence of perinatal depression and its determinants in Mainland China: a systematic review and meta-analysis. J Affect Disord. (2020) 277:1022–37. doi: 10.1016/j.jad.2020.07.046 33065811

[24] Sun G Wang Q Lin Y Li R Yang L Liu X . Perinatal depression of exposed maternal women in the COVID-19 pandemic in Wuhan, China. Front Psychiatry. (2020) 11:551812. doi: 10.3389/fpsyt.2020.551812 33391042 PMC7772463

