# Supplementary material for: Prenatal depression exposure and infant developmental outcomes: a retrospective cohort study of reduced fetal growth indicators, elevated neonatal heart rate, and developmental trajectories in China
Source: Front Psychiatry. 2026 Jun 22;17:1827974. doi: 10.3389/fpsyt.2026.1827974 (PMC13333675; doi:10.3389/fpsyt.2026.1827974)
Supplement: Supplementary file 1 [file Supplementaryfile1.docx]

**Supplementary Figure 1.** Love plot assessing covariate balance before and after propensity score matching.


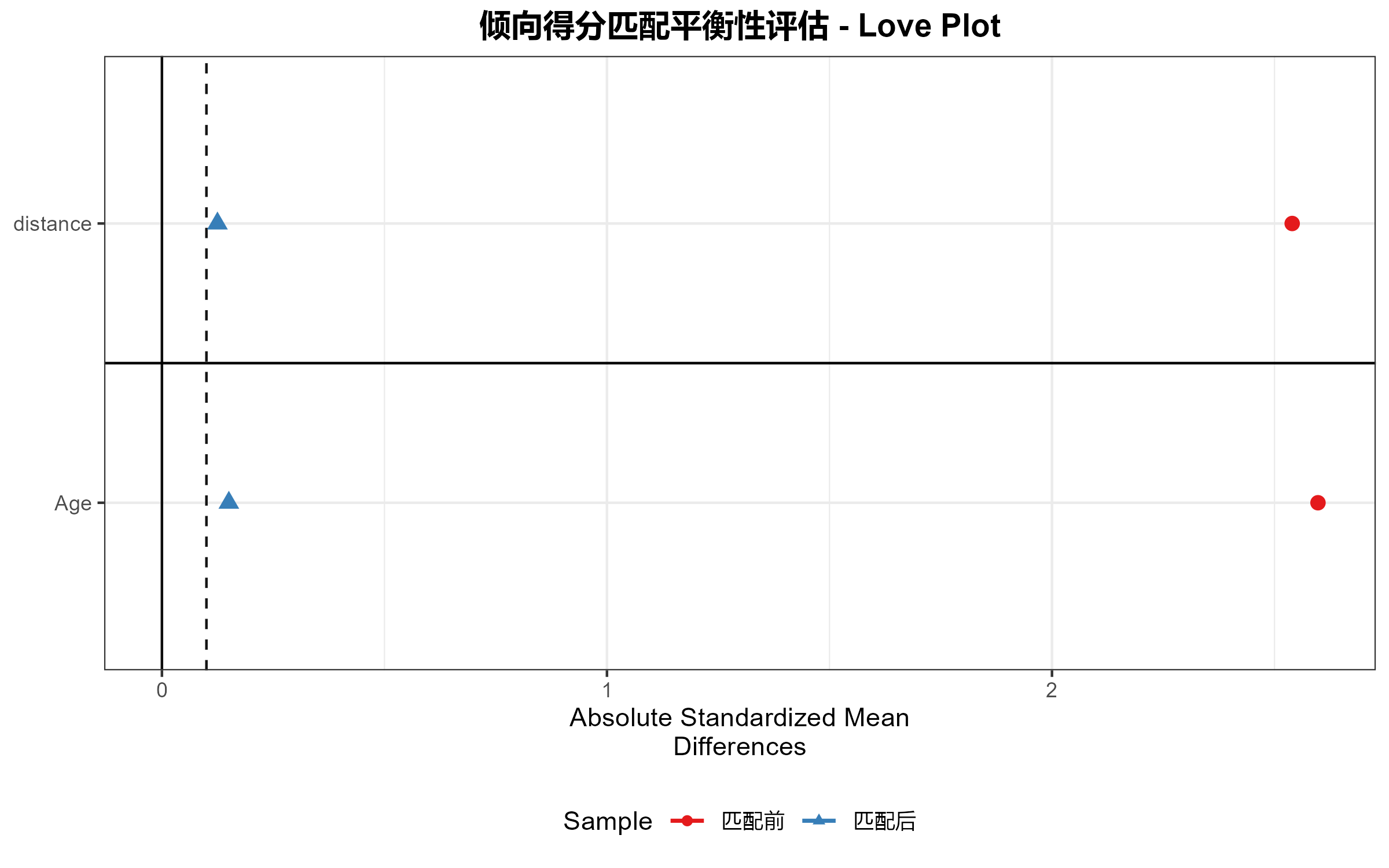


This plot visualizes the standardized mean differences (SMDs) for the matching covariate (Age) between the treatment and control groups. Each point represents the absolute SMD for Age. The red circles indicate the SMD in the unmatched original sample (SMD = 2.70), showing severe initial imbalance. The blue triangles show the SMD in the matched sample (SMD = 0.12) after 1:1 nearest-neighbor propensity score matching, demonstrating a substantial improvement in balance. The vertical dashed gray line marks the commonly recommended threshold for good balance (SMD < 0.1). Although the post-matching SMD slightly exceeds this threshold, the dramatic reduction confirms the effectiveness of the matching procedure in mitigating age-related confounding.

**Supplementary Figure 2.** Love Plot: Covariate Balance Before and After Propensity Score Matching


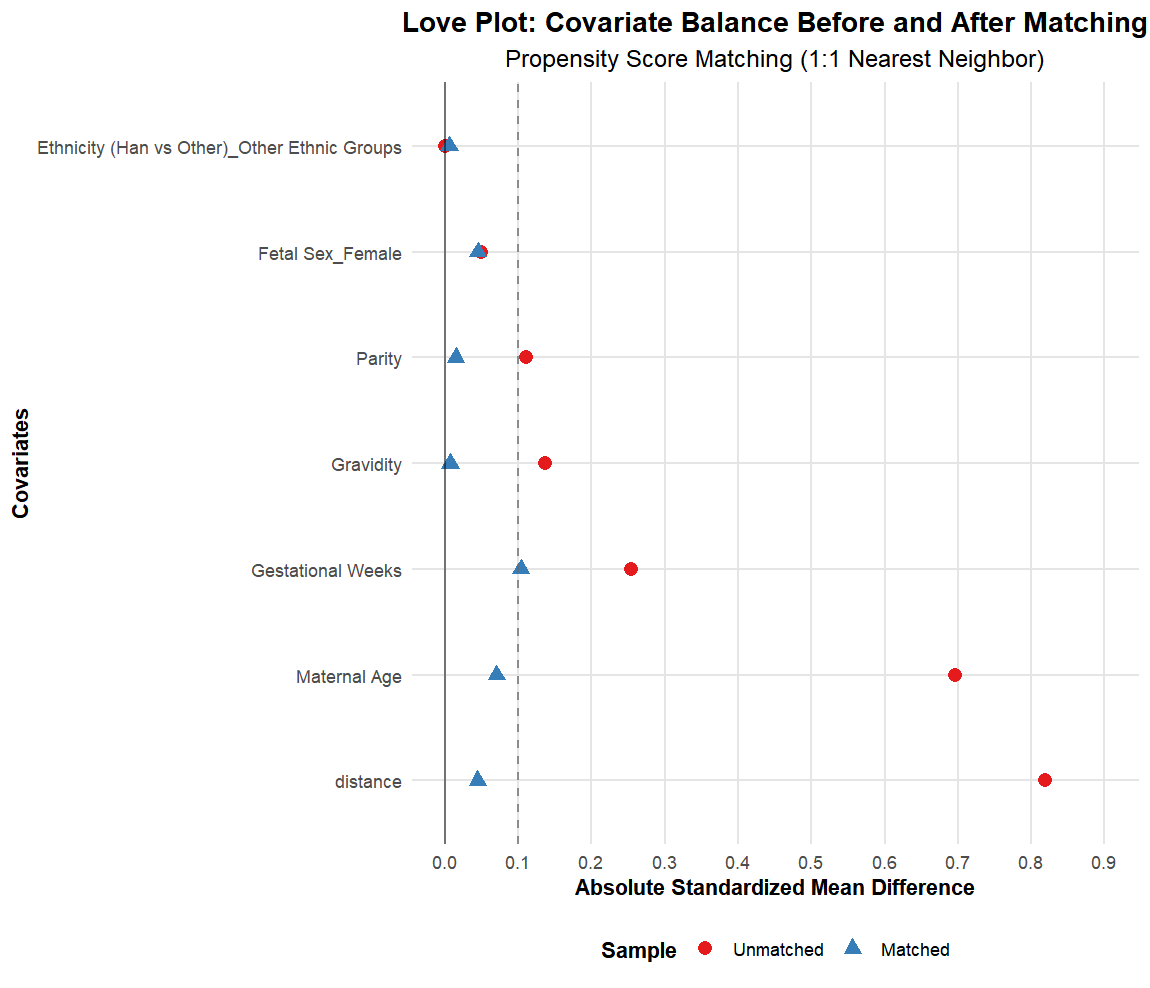


The Love plot displays the absolute standardized mean differences for covariates included in the propensity score model before (● red circles) and after (▲ blue triangles) 1:1 nearest neighbor propensity score matching. Covariates included maternal age, ethnicity (categorized as Han Chinese or Other Ethnic Groups), gravidity, parity, fetal sex, and gestational age. The dashed vertical line represents the threshold of 0.1 for standardized mean differences, below which covariates are considered well-balanced between groups.
